# Self-Consciousness and Self-Awareness: Associations between Stable and Transitory Levels of Evidence

**DOI:** 10.3390/bs13020117

**Published:** 2023-01-30

**Authors:** Maurício Majolo, William Barbosa Gomes, Thiago Gomes DeCastro

**Affiliations:** Institute of Psychology, Universidade Federal do Rio Grande do Sul, Porto Alegre 90035-003, Brazil

**Keywords:** self-awareness, self-consciousness, assessment, experience sampling, mixed methods

## Abstract

The theory of objective self-awareness predicts the assessment of stable or dispositional self-consciousness and transitory or situational self-awareness. The aim of the present research was to investigate potential associations between patterns of experiential self-description to scores on self-report measures of dispositional self-consciousness. A total of 64 Brazilian volunteers (M_age_ = 29.7, SD = 8.79, 64.1% female) responded to the Revised Self-Consciousness Scale, the Philadelphia Mindfulness Scale, and the Rumination–Reflection Questionnaire before participating in an experience sampling protocol. The protocol consisted of random daily requests for up to four self-description experiences across seven consecutive days. Participants recorded audio messages on their mobile phones in reply to each request describing their current experience. Reports were analyzed through a reflexive thematic analysis that produced twenty sub-themes accounting for descriptive markers of experience. Based on those descriptive markers, the qualitative data were then transformed into quantitative data for the situational self-awareness indexes. Evidence of association between self-consciousness and self-awareness was stronger for the awareness subscale in a positive correlation with affective situational self-awareness and in a negative correlation with mental representational transitory self-awareness. Although relational evidence has been provided, the data reinforced the theoretical distinction between self-consciousness and self-awareness.

## 1. Introduction

Objective self-awareness has been defined as the human ability to become the object of its own attention, actively identifying, processing, and storing information about the self [1]. In line with this tradition, an early distinction between self-consciousness and self-awareness was proposed to specify what a trait self-consciousness measure was purposely assessing [2]. Such distinction informed a conceptual division between a dispositional form of self-consciousness and situational self-awareness, which remains determinant for the general field of self-awareness research. The dispositional self-consciousness construct (DSC) refers to a stable trait of personality resultant of an individual history of self-focusing operationalization and developmental sediment. On the other hand, situational self-awareness (SSA) refers to a transient state of self-focused attention dependent on the control of the environment stimulation [3].

Currently, a considerable range of self-report scales aimed at measuring DSC is found in the literature [4,5,6]. The different conceptual factors of these instruments are researched and updated continually over time, composing a complex panorama of DSC assessment. In mental health research, for example, the association between DSC and personality, well-being, psychosocial markers, and specific mental disorders is well documented [7,8,9,10,11].

In its turn, the SSA research picture is particularly different. Fewer self-report measures were developed [3,12], and the attempts to adapt situational self-awareness scales did not provide satisfactory psychometric evidence across different cultures [13,14], indicating a potential inherent difficulty in measuring the situational variable. However, research on SSA has increased within Neurosciences. Specific neurobiological correlates of SSA have been evidenced from the methodological intersection between neuroimaging techniques and first-person approaches in experimental settings. SSA has been found to be associated with the existence of a high-level multisensory and premotor processing centered in the central insula that interconnects diverse networks, such as those related to the sense of agency, sense of self-ownership, decision-making, and body schema [15,16,17,18]. SSA has also been found to mediate the correspondence between structures of the medial prefrontal cortex, posterior cingulate cortex, and precuneus with executive functions of self-regulation, self-reflection, body image, theory of mind, and autobiographical memory, for example [19,20,21,22].

In this diverse set of evidence, there have been propositions of a conceptual framework that sought to integrate the greatest number of variables arising from pioneering studies on self-awareness in a coherent translational model [23,24]. Such a model can serve as a translational theory for different scientific disciplines. Its broad scope includes research from neurobiological correlates and developmental aspects to cognitive processes and social/environmental features related to self-awareness. Such a focus would mostly corroborate research on the mediational cognitive mechanisms of the transient self-referring experience, especially self-talk [25,26,27]. However, important epistemological issues, such as the exact theoretical and empirical relationship between the different dispositional and situational constructs of self-awareness, as well as the methodological challenge of including its well-known pre-reflexive and phenomenal aspects [28], could be fundamentally neglected.

In this view, it would be problematic that self-reports and questionnaires emerge as the main methodological strategy for assessing stable and transient self-focused attention. On examining the possible theoretical overlapping across different self-consciousness self-report measures, evidence has pointed to two general epistemological features within different DSC measures [29]. Results evidenced a strong overlapping focus referring to the evocation and evaluation of the content of thoughts and memories, corresponding to a dispositional self-consciousness. Yet another focus emerged in the data referring to the phenomenality of the experience itself, embodied and situated, corresponding to situational self-awareness; these data were restricted to a subcomponent of awareness mindful attention. Such duality not only reinforces the temporal dimension that operates in the distinction between notions of DCS (past) and SSA (present), but also the fundamental participation of a pre-reflective and experiential dimension in the individual capacity of self-focus.

Alternative methodological strategies to assess SSA are known in the literature, with a major emphasis on ecological protocols of experience sampling. Experience sampling techniques were initiated with portable communication tools, such as pagers and smartphones, and involved collecting repeated responses randomly over days or weeks with participants in their natural environments [30]. Descriptive Experience Sampling (DES) is one experience sampling proposal characterized by open descriptions of the participants’ experiences at random moments. The experience reports are given preferably by speaking out loud about their current experience, followed by subsequent interviews to approximate a reliable interpretation of the reports [31]. A second experience sampling proposal is the Experience Sampling Method (ESM), in which closed questions and self-report Likert-type scales are applied to capture the experiences lived by the participants since the last response request. Contrasting with the more descriptive phenomenological root of DES, the ESM aims to obtain a representative and quantified sample of experiential markers from a pre-specified population sample [32].

Literature applying DES points to a growing field covering topics mainly related to inner speech [33,34,35,36]. Such investigations were able to observe that inner speech and thinking aloud may serve as active tools of self-awareness for facilitated access to the flow of thoughts, even allowing for the apprehension of mind-wandering episodes [37]. Other self-related phenomena and cognitive architecture matters, in addition to inner speech topics, are investigated through the application of non-descriptive approaches, such as the ESM [38,39,40].

Investigations aiming at potential associations between situational and dispositional levels of self-awareness are scarce and could benefit from experience sampling protocols. Previous work that explicitly investigated the association between stable and transitory evidence for DSC and SSA found that lower levels of private self-consciousness were, in fact, correlated to descriptive positive self-focused attention and less ruminative thinking [41]. However, the lack of proper technological tools to capture verbalization aloud and an experience sample protocol limited to two days prevent these results from being considered ecologically valid. Moreover, self-consciousness measures were limited in the period of the publication.

Hereupon, coupling modern experience sampling protocols with robust self-report measures of self-consciousness could be a promising methodological alternative. First, temporal dissociation between current conscious experience and self-observations [42] could be restricted by applying experience sampling techniques. Second, translational dissociation could be limited by associating experiential self-descriptions with dispositional indexes provided by robust self-report measures.

In the present study, we investigated whether patterns of experiential self-description obtained through DES could be associated with dispositional indexes of self-consciousness, self-rumination, self-reflection, and awareness. When the association was detected with any of the DSC scales, we tested whether groups of high and low DSC could differentiate SSA distribution. Moreover, we explored if self-report mental health issues and related behavior, along with sociodemographic characteristics, could impact the variance of DSC and SSA indexes. In order to enable the test for DSC and SSA association, self-descriptions provided by DES were transformed into self-description coefficients of SSA.

## 2. Materials and Method

### 2.1. Participants

We collected data from a sample pool consisting of 254 volunteers (M_age_ = 30.73, SD = 9.07, Range = 18–52 years old, 65.7% female) that responded online to the DSC measures and a sociodemographic and mental health protocol. Participants were recruited online through the authors’ university social media channels. From this pool, 67 participants randomly recruited took part in the experience sampling protocol, but three were excluded for data analysis due to not responding the protocol requests for at least one day. Final sample consisted of 64 participants (M_age_ = 29.70, SD = 8.79, 64.1% female). Data collection was fully performed online over the course of 2021−2022 academic year due to the ongoing COVID-19 pandemic. Sample size power analysis computation was performed to estimate whether a bivariate correlation analysis with 64 participants could predict adequate statistical power. For a mean association between variables of 0.33 (*p* < 0.05), the sample size can predict a power (1-*β* err probability) of 0.85 (G*Power, version 3.1.9.7).

### 2.2. DSC Measures

All DSC measures were responded online through the Google Forms platform. Note that all Cronbach alpha values reported here are those obtained for the present sample.

#### 2.2.1. Revised Self-Consciousness Scale

The Revised Self-Consciousness Scale (RSCS) [43] is a 22-item Likert-type measure composed of three factors: private self-consciousness (PrSC; 9 items; sample item: “I think about myself a lot”), public self-consciousness (PuSC; 7 items; sample item: “I’m concerned about what other people think of me”) and social anxiety (SA; 6 items; not used in the research). The version applied in the current research was the Brazilian adaptation of RSCS [44]. The Cronbach alphas obtained for the present sample were 0.69 for PrSC and 0.77 for PuSC. The social anxiety subscale was not administered to the sample since it was not part of the self-focused attention research problem.

#### 2.2.2. Rumination–Reflection Questionnaire

The Rumination–Reflection Questionnaire (RRQ) [6] is a 24-item Likert-type measure composed of two factors that distinguish motivational and attentional facets of private self-consciousness: Reflection (Ref; 12 items; sample item: “I love exploring my ‘inner’ self”) and Rumination (Rum; 12 items; sample item: “Often I’m playing back over in my mind how I acted in a past situation”). The version applied in the current research was the Brazilian adaptation of RRQ [45]. The Cronbach alphas obtained for the present sample were 0.89 for Ref and 0.92 for Rum.

#### 2.2.3. Philadelphia Mindfulness Scale

The Philadelphia Mindfulness Scale (PMS) [46] is a 20-item Likert-type measure composed of two factors that assess: a sustained attention towards the self in the present moment named awareness (Awa; 10 items; sample item “I am aware of what thoughts are passing through my mind”), and an openness, curiosity and acceptance attitude towards experience named *Acceptance* (Acc; 10 items; not used in the research). Even though the awareness subscale intends to assess a present-moment experience, previous research has defined the scale as a dispositional measure of self-consciousness for its instruction to consider the last two weeks as a time framework for the response to the scale items [29]. The version applied in the current research was the Brazilian adaptation of PMS [47]. The Cronbach alpha obtained for Awa in the present sample was 0.85. The Acc subscale was not administered to the sample since it was not part of the self-focused attention research problem.

### 2.3. Sociodemographic and Mental Health Questionnaire

The Questionnaire was responded online through the Google Forms platform and was composed of two components. The first comprised the participants’ sociodemographic information, including their age, sex, religious practice, education status, marital status, area of academic training, place of residence, work occupation, number of cohabitants, and number of children. The second comprised the participants’ mental health information, including whether they were previously diagnosed with any mental disorder by a mental health professional, used continuous psychotropic drugs, were or have been in a psychotherapeutic process, had or have had a habit of keeping some kind of personal diary, and if they had current experience with some kind of meditation practice.

### 2.4. Experience Sampling

#### 2.4.1. Training

After completing the responses to the DSC measures and the questionnaire participants were invited to participate in the Experience sampling protocol. For those who participated in this stage, a web-conference training was scheduled to instruct participants on the duration, objective of the protocol, and *smartphone* usage during the experience sampling responses. The individual training followed a textualized and rigorous script, with an average duration of one hour, through which participants received the same guidelines for installing and using the *Telegram* instant messaging app along with instructions regarding the data collection procedures. In this step, participants were able to solve any doubts regarding the procedure with the researcher practicing examples of situational self-descriptions.

The training sought to instruct the participants with notions of phenomenological description so that their answers were as close as possible to their current and spontaneous experience. For systematic scripting of the phenomenological skills aimed at this research, a theoretical scheme was applied to differentiate descriptions with content of current experiences from non-current experiences [48]. Examples of current experience contents would be the identification of what was being experienced at that present moment (“Object of experience”), description of smaller objects that make up the total object experienced (“Object characteristics”), description of the appearance of the object in perceptual qualities (“Noematic characteristic”), description of phenomenological qualities of the object’s mode of presentation (“Noetic characteristic”), description of the intentional act of revealing the object to consciousness (“Correlative characteristic”), and/or description of non-object characteristics experience that do not fit into the previous categories (“Other”). Likewise, the research’s lack of interest in non-current experience content was explained, such as description of thoughts, memories, imaginations, or emotions induced by or derived from the perceptive experience of an object (“Associations”), casuistic explanation of the experience in reference to subpersonal processes (“Subpersonal Explanation”), and anticipation of general claims about reality based on the experience being described (“Generalization”).

#### 2.4.2. Data Collection Procedures

For the data collection, the participants had to constantly carry their personal *smartphone* for a period of seven consecutive days. During this period, the *smartphone* should be in good working order with a stable internet connection and adequate battery charge between 10 am and 8 pm. The *Telegram* messaging app installed and verified in the training procedure should be activated during the whole collection period.

Based on the DES procedure [31], the protocol applied over seven days had four daily response requests, triggered automatically by a pre-programmed resource and made available for direct and free use within the *Telegram* platform. The content of the automatic request for response message was always the same: “What was in your experience just before you read this message?” [49]. The message shots took place at random times within each of four daily blocks (10:00 am–12:00 am, 2:00 pm–4:00 pm, 4:00 pm–6:00 pm, and 6:00 pm–8:00 pm) and separated by at least one hour each. Responses should be made in audio format with a maximum length of 120 s without a minimum duration. In each message, participants were instructed to observe the current experience interrupted by the notification of the request in immediate retrospection, describing aloud and spontaneously the salient aspects of their most recent stream of consciousness.

It was not necessary for the participant to respond as soon as a request was sent to their *smartphone*, as long as they responded as soon as they had seen the message. In case the participant could not respond right after viewing the message, in the interest of avoiding any physical accident, interpersonal or work conflict, or exacerbation of mental or moral suffering, the participant was instructed to allow himself/herself to forget about the request and respond to it as if he/she had read it for the first time when remembering, spontaneously and if still in time, in a more favorable future context. In the event of late requests accumulating, for whatever reason, it was advised that all were disregarded, with the exception of the most recent one, which would then be answered.

### 2.5. Ethical Considerations

This study was conducted following the Declaration of Helsinki tenets and ethical standards for research involving Humans, Ministry of Health, Brazil. The protocol was approved by the Ethics Committee of authors’ university.

#### Data Preparation and Analysis

DSC data and Questionnaire information were tabulated in SPSS 26 to generate descriptive statistics and normality plots. Then the audio recordings from the experience sampling protocol were transcribed in separate *word* files, one for each participant, in chronological order of responses. The individual average response duration of the audio recordings was integrated into the SPSS dataset.

For the initial qualitative analysis of the audio recordings, data from ten randomly chosen participants were exported to NVivo (version 1.5), and a reflexive thematic analysis [50] was conducted. The inductive process was oriented by six steps: (1) familiarization with the data, (2) systematic coding, (3) generation of initial themes (code clusters), (4) review and conceptualization of initial themes, (5) refinement and naming of final themes, and (6) analysis report writing. This analysis generated five general experience self-description themes: time, direction, form, sense, and valence. Each theme comprised subthemes that resulted in 20 situational self-focused attention descriptors (Table 1).

The descriptors were then applied by two independent judges to all the qualitative data with the intent to generate situational self-description coefficients (SSA). This procedure is implied in examining each participants’ response applying the 20 situational self-description grid in binary logic. Codes of “0” (absent) or “1” (present) were applied to each of the 20 situational self-descriptor markers in regard to each audio recording. Then a sum of all classifications for each subtheme was divided by the number of total responses of each participant to generate a specific situational self-description coefficient. Each participant then had a total of 20 situational self-description coefficients based on the 20 subthemes extracted in the thematic analysis.

To assess whether the judges agreed with the data classification, an intra-class correlation coefficient (ICC) was performed, resulting in a 0.955 agreement score with a 95% confidence interval. Considering the high level of agreement between the two judges, a simple mean between the judge’s scores was computed to generate the final 20 situational self-description coefficients. These coefficients were then integrated into the SPSS dataset.

Mann–Whitney and Kruskall–Wallis tests were initially conducted to compare DSC and SSA indexes between sociodemographic and mental health categories. Then partial non-parametric correlations were computed, controlling for the effect of specific sociodemographic and mental health information, between DSC and SSA indexes. Finally, Mann–Whitney tests considering high and low DSC profiles were conducted, taking SSA indexes as dependent variables.

### 2.6. Sociodemographics, Mental Health and DSC/SSA

The sample comprised different respondent profiles in terms of sociodemographic and mental health self-reported information (Table 2). Several sociodemographic variables and mental health issues impacted the mean distribution of DSC and SSA indexes.

Sociodemographic variables that impacted the mean distribution of DSC variables were marital status and religious practice. Participants who had single marital status presented higher scores on the PrSC scale (*U* = 189.000, *z* = −2.389, *p* < 0.0117, r = 0.030), and those who stated that they did not identify with any religion also showed higher scores on the PrSC scale (*U* = 318.500, *z* = −2.336, *p* < 0.019, *r* = 0.29). In turn, mental health aspects that impacted the mean distribution of DSC variables were mental disorder diagnosis, psychotherapy status, and meditation practice. Ruminative thinking (Rum) was more prevalent among participants without a mental disorder diagnosis (*U* = 170.500, *z* = −2.155, *p* < 0.031, *r* = 0.27). For participants who were undergoing psychotherapy, higher PrSC scores (*H* = 9.077, *p* < 0.0111) and Ref scores (*H* = 9.106, *p* < 0.0111) were observed in comparison to the other groups. Similar results were observed in meditation practice, with practitioners presenting higher scores on PrSC (*H* = 9.775, *p* < 0.0124) and Ref (*H* = 18.402, *p* < 0.0101).

In its turn, sociodemographic variables that impacted the mean distribution of the self-descriptive markers of SSA were the participants’ age and gender, marital status, having children, and religious practice. Younger participants scored higher on self-regulation SSA (*U* = 299.500, *z* = −2.856, *p* = 0.004, *r* = 0.36) and self-reflection SSA (*U* = 317.0, *z* = −2.621, *p* = 0.009, *r* = 0.33). Women had higher scores on the SSA temporal variables of past SSA (*U* = 320.500, *z* = −2.006, *p* = 0.045, *r* = 0.25) and present SSA (*U* = 302.500, *z* = −2.256, *p* = 0.024, *r* = 0.28). Participants with a single marital status had higher scores on self-regulation SSA (*U* = 134.0, *z* = −3.299, *p* < 0.0101, *r* = 0.41) and self-reflection SSA (*U* = 170.500, *z* = −3.299, *p* = 0.007, *r* = 0.41). The results were similar when comparing participants with and without children, indicating that those without children had higher scores on self-regulation SSA (*U* = 155.500, *z* = −2.940, *p* = 0.003, *r* = 0.37) and self-reflection SSA (*U* = 176.500, *z* = −2.586, *p* < 0.011, *r* = 0.32). Participants who stated that they did not identify with any religion showed higher negative valence SSA scores (*U* = 344.500, *z* = −1.970, *p* = 0.049, *r* = 0.25). Regarding the mental health information and SSA, participants who reported not using psychotropic drugs had higher Non-Symbolic thought SSA scores (*U* = 135.000, *z* = −2.174, *p* = 0.03, *r* = 0.27). For participants who were undergoing psychotherapy, higher Private SSA scores (*H* = 10.103, *p* = 0.006), Public SSA (*H* = 11.152, *p* = 0.004), and Sensation SSA (*H* = 9.390, *p* = 0.009) were observed. As for those who practiced or had already practiced meditation, the highest scores for the External World SSA variable (*H* = 6.131, *p* = 0.047) were observed. Finally, those who had already kept a personal diary had higher scores on the self-regulation SSA (*H* = 6.711, *p* = 0.035) than those who had never kept a diary.

### 2.7. Association between DSC and SSA

The impact of sociodemographic and mental health self-reported information over DSC and SSA indexes mean distribution implied that any correlation between dispositional and situational self-awareness should consider the different participant profiles. In this direction, all the following variables were controlled in a partial correlation between DSC and SSA indexes: age, sex, marital status, children, religious practice, mental disorder diagnosis, psychotropic drug use, psychotherapy status, meditation practice, and keeping a personal diary. When controlling for these variables, some correlations could be observed between DSC and SSA indexes (Table 3).

Among the SSA indexes, Sense experience-function indexes were the more correlated coefficients with other SSA indexes. For the self-regulation SSA index, correlations were found with Private SSA (*ρ* = 0.34, *p* < 0.015), Sensation SSA (*ρ* = 0.45, *p* < 0.001), and Non-Symbolic thought SSA (*ρ* = 0.38, *p* < 0.011). For the self-reflection SSA index, correlations were found with Private SSA (*ρ* = −0.33, *p* < 0.015), Public SSA (*ρ* = 0.41, *p* < 0.011), Undefined form SSA (*ρ* = 0.33, *p* < 0.015), Verbal Thinking SSA (*ρ* = 0.32, *p* < 0.015) and Observation SSA (*ρ* = −0.42, *p* < 0.011). For the Pragmatic SSA index, correlations were found with Affect SSA (*ρ* = −0.49, *p* < 0.0101), Non-Symbolic thought SSA (*ρ* = 0.28, *p* < 0.015), Observation SSA (*ρ* = −0.35, *p* < 0.011), positive valence SSA (*ρ* = −0.42, *p* < 0.011) and neutral valence SSA (*ρ* = −0.47, *p* < 0.0101). Finally, for the Observation SSA index, correlations were found with Private SSA (*ρ* = 0.28, *p* < 0.015), Sensation SSA (*ρ* = 0.44, *p* < 0.0101), Affect SSA (*ρ* = 0.69, *p* < 0.002), positive valence SSA (*ρ* = 0.54, *p* < 0.0101), negative valence SSA (*ρ* = 0.44, *p* < 0.0101) and neutral valence SSA (*ρ* = 0.35, *p* < 0.011).

The mean duration of responses to the experience sampling protocol correlated strictly with some SSA indexes. The significant associations found between the mean duration of responses and SSA were with the Public SSA index (*ρ* = 0.37, *p* < 0.011), the Sensation SSA index (*ρ* = 0.32, *p* < 0.011), the Mental representation SSA index (*ρ* = 0.28, *p* < 0.015), the Non-Symbolic thought SSA index (*ρ* = 0.30, *p* < 0.015), the self-regulation SSA index (*ρ* = 0.39, *p* < 0.011), the self-reflection SSA index (*ρ* = 0.41, *p* < 0.011), and the Mixed valence SSA index (*ρ* = 0.28, *p* < 0.015).

### 2.8. Differences between DSC Profiles

Comparison between groups based on the PrSC subscale evidenced that participants with higher scores according to the subscale had lower scores in Mental representation SSA (*U* = 341.500, *z* = −2.291, *p* = 0.022, *r* = 0.29) and Mind Wandering SSA (*U* = 348.500, *z* = −2.314, *p* = 0.021, *r* = 0.29). For the Rum subscale, participants with lower scores had higher scores in Verbal Thinking SSA (*U* = 303.0, *z* = −2.759, *p* = 0.006, *r* = 0.34). Comparison between groups considering the variable Awa (Table 4) evidenced that participants with higher awareness scores had higher scores in Affect SSA (*U* = 324.500, *z* = −2.513, *p* = 0.020, *r* = 0.31) and positive valence SSA (*U* = 322.0, *z* = −2.550, *p* = 0.011, *r* = 0.32). On the other hand, these same participants had lower Mental representation SSA scores (*U* = 296.0, *z* = −2.904, *p*= 0.011, *r* = 0.36) and neutral valence SSA scores (*U* = 315.0, *z* = −2.642, *p*= 0.008, *r* = 0.33).

## 3. Discussion

The results partially satisfy the general objective of the research, which aimed to find associations between DCS and SSA. The associations found were mediated by several aspects of sociodemographic and mental health profiles. In this sense, the few associations indicated that even with variation in stable and transient self-awareness evidence between different population profiles, it is possible to affirm the existence of some level of relationship between the two modalities of self-awareness. However, when such associations are examined, there is a tendency for the situational variables to cluster around the DSC variable awareness.

Previous research [29] had already observed a difference in the distribution of the awareness measure in relation to other variables of dispositional self-consciousness, such as private self-consciousness, self-rumination, and self-reflection. This result was interpreted as a differential theoretical brand between self-awareness as an object of reflective access to past experiences (i.e., DSC) or as a pre-reflexive feature of the unthematized apprehension of one’s experience in the present moment (i.e., SSA). Data from the present research corroborate this differentiation by indicating a greater number of associations between SSA variables and awareness of DSC. It should be noted that the measure refers to an awareness index within mindfulness literature aimed at full attention to the current moment of the experience. In this sense, even being operationalized as a trait self-report measure, it is possible that this dispositional dimension refers precisely to the individual’s stable tendency or the ability to apprehend situational experience, bringing it closer to the definition of transitory self-awareness.

By specifically observing the six variables significantly associated with the awareness subscale, it is possible to better identify the characteristics of the situational experience linked to the trait that the subscale measures. Firstly, the positive correlations with both PrSC and PuSC reinforce that awareness does not distinguish the direction of the experience’s self-focus attention, fundamentally referring to its phenomenological characteristics, regardless of the direction of the attentional process. Second, the absence of significant associations with the RRQ subscales reinforces the dissociation of the awareness construct with psychological qualities of mental health adaptability and with subjective elements of judgment about the valence and function of individual experience. Third, the positive correlations with Affect SSA and positive valence SSA and the negative correlations with Mental representation SSA and Neutral valence SSA denote that the more frequent were the descriptions of experiences in the form of emotions, feelings, and moods, as well as experiences with global affective quality of positive valence, the higher the awareness scores. On the other hand, the more frequent the descriptions of experiences in the form of mental representations, as well as effectively disconnected experiences, the lower the awareness scores.

It is necessary to remember that although the awareness construct of the scale refers to the tendency or ability to disinterested, non-explanatory, and non-evaluative access to situational experience, it is not necessary that the experience being observed and described in the present is effectively null. On the contrary, what the results suggest is that the trait measured by awareness is more effective in spontaneously detecting effects, especially positive ones, than neutral experiences. Furthermore, mental representations, which are usually elicited and sustained by syntactic-semantic structures parallel to the immediate perception of experience [51], quite unlike affections, seem to be less observed and described by people with a higher level of awareness.

Following the thesis that the Mental representation SSA index can be presented as statistically antithetical to awareness, it was observed that it also correlated negatively with PrSC and positively with the Verbal Thinking SSA index and average duration of the audios. These results suggest that a greater occurrence of descriptions of mental representations seems to be associated with greater difficulties in describing private self-aspects. Likewise, the more limited the pre-reflective access to experience and the greater the lack of objectivity in the description of the private phenomena themselves, the more extensive the reports tended to be. In cases where the subject may have had difficulty producing a phenomenological description of their experience, subjunctive and allegorical modes of verbal expression are often used similarly to mental representations as associative reasoning tools [49]. In these cases, the subject suggests to the evaluator of his/her report the experience of a verbal thought or an internal conversation that, if it is actually present in his/her experience, possibly stems from the question and not immediately prior to the experience sampling protocol question.

Two of the SSA indexes with experience function were also positively associated with the mean duration of responses, in a moderately strong magnitude, namely self-regulation, and self-reflection. There are two possible explanations for these associations. The first is the assumption that people with greater self-regulation and self-reflection in the descriptions of experiences would spend more time in their days occupied with aspects of their own self and would naturally have greater fluency and ease in offering detailed descriptions of their private phenomena. This explanation is difficult to assess since the accuracy of the reports is neither verifiable nor objectified by the method. Therefore, it is equally plausible the hypothesis that a considerable portion of these occurrences is due to the opportunity taken from the response request to elaborate and elucidate on aspects of the self that were not present in the interrupted experience. As can be seen, the two hypotheses are not mutually exclusive and seem to be influenced by the dispositional characteristics of each subject.

As for the use of sociodemographic variables and mental health information, the results not only illustrate their duly controlled influence on correlation tests but also lead to the speculation of interesting theoretical hypotheses. To begin with, higher levels of self-regulation SSA and self-reflection SSA were found for the youngest portion of the sample, likewise for those who had never been married and for those who had no children. These data allow for future investigations that address these SSA differences in more specific contextual and developmental settings. The literature already indicates, for example, that adults have progressive losses in metacognitive skills over the years [52] but also that older adults have more positive attitudes toward their own mental health [53].

Non-religious people had higher negative valence SSA scores, corroborating the extensive evidence of an association between religiosity and well-being [54], along with higher PrSC scores. The literature comparing measures of self-consciousness and religiosity is scarce, and considering the different existing religious traditions and practices along with the different possible levels of personal involvement, it seems more cautious to say that the difference found for PrSC in the present sample, in which most religious participants were Christians, suggests further investigations with more diverse religious samples.

Regarding the diagnosis of a mental disorder by a health professional, the difference found is counterintuitive. People without a diagnosis had higher Rumination scores. The finding contradicts the literature [55,56] and possibly indicates sample bias. However, considering the low magnitude of the different sizes, the fact that all participants with the diagnosis were also in psychotherapy and/or made continuous use of psychotropic drugs can help to understand the result. Corroborating this hypothesis, the comparison between psychotherapy groups showed that people who had never been in psychotherapy and people who had been in psychotherapy but not at that time had lower PrSC, self-reflection (RRQ), SSA Private self-focused attention, and SSA Public self-focused attention scores than people who were in psychotherapy. However, there was no significant difference between people who had never been in psychotherapy and people who had been but not at that time, suggesting that possible gains from psychotherapy that could influence the aforementioned DSC and SSA scores were lost or attenuated over time.

For the meditation practice, people who were engaged in some regular practice had higher PrSC scores than those who discontinued or had never practiced. This finding indicates that the increase in dispositional attention to private self-aspects possibly only happens while individuals have a systematic practice of self-observation in their routine since this difference is not found between people who interrupted the practice and those who never meditated. It is different in the case of the Ref subscale, in which the group that used to meditate but not at that moment obtained higher scores than people who never meditated. The evidence raises the hypothesis that the trait measured by the Ref subscale predates the practice of meditation and that people with higher levels of self-reflection seek this form of activity.

The research is exploratory by nature and presents methodological and sampling limitations. First, all 64 participants who participated in the experience sampling had at least an incomplete higher education degree, configuring a general population subsample. Given this characteristic and the nature of convenience sampling, it is safe to assume that those interested in the research showed scores and patterns of DSC and SSA not representative of the general population profile of the country where the study was conducted. Furthermore, it is relevant to note that social and linguistic characteristics particular to a Brazilian sample might have held an influence on the results since situational self-awareness is especially affected by linguistic patterns of expression and social features specific to participants’ culture. Acknowledging this potential bias is important when transposing these results to other cultures. In addition, a prototypical phenomenological training script was used, not experimentally validated, given the lack of suitable models for replacing phenomenological interviews in descriptive experience sampling protocols. Studies on phenomenological skills training certainly have a long way to go, and the method employed in the present research is an important step in this direction, in addition to the indications made for greater attention to instruments and theoretical notions associated with awareness in mindfulness literature.

It is suggested that the thematic analysis procedure that generated the SSA self-descriptive markers, built especially for the present research, be replicated and reproduced, enabling, through the accumulation of evidence, a robust protocol for evaluating experience descriptions. The discussion of the results of this study raised a series of theoretical hypotheses that suggest research questions and sampling criteria for future studies and provide an opportunity to verify the method applied in the present study. Regarding the quantitative analysis procedures, it is important to note that the use of medians to separate the sample into groups for the test of comparison of means sacrifices the sensitivity of the instruments applied to measure the variables. It is recommended that future research gather larger sample sizes for division into more homogenous groups or use naturally categorical variables or cutoff points for continuous variables indicated in the literature.

In general, the association between stable and transient levels of evidence of self-awareness was clarified, reinforcing and detailing the difference in epistemological access between notions of a dispositional self-consciousness pertaining to a functionalist tradition of social psychology and personality and the notions of situational self-awareness drawn from experiential, psychological traditions. However, possible approximations were also presented through the awareness subscale to descriptors of SSA. Evidence suggests that self-consciousness scales investigate epistemic, attentional, and adaptive characteristics more related to a notion of self-concept as a stable reflective product of self-awareness [57]. At the same time, evidence from SSA categories indicates that the experiential and instrumental aspects of self-awareness are perhaps better addressed by other constructs and methods, not directly linked to the nomenclature and operationalization proposed by the Theory of Objective Self-Awareness [1].

## Figures and Tables

**Table 1:** Descriptive markers for qualitative analysis of self-descriptions of experience.

Themes (Nature)	Subthemes Descriptive Marker	Guide-Question: “Does the Experiential Description Clearly Refer to…
Time	Past	…an experience reported in past grammatical tenses?”
	Present	…an experience reported in present grammatical tenses?”
Direction	External world	…aspects of publicly observable objects, external to the subject, such as events in time and space, or the appearance of things, reported as though independent of first-person perspective?”
	Private	…aspects internal to the subject and only observable by him, such as characteristics of his/her own personality or the experiences he/she reports?”
	Public	…aspects of the subject himself/herself that define him/her as a social, publicly observable object, such as his/her own appearance, his/her behavior and ‘way of being’, his/her interactions with other people, or the perception he/she infers from others about himself/herself?”
Form	Undefined	…an experience that possibly has form, but for which the subject, for whatever reason, does not use specific or sufficient words or explanations to define?”
	Sensation	…stimuli perceived by the body’s senses, whether exteroceptive, proprioceptive or interoceptive?”
	Affect	…emotions, feelings or moods, regardless of cause or complexity?”
	Mental representation	…sensory and/or affective aspects of objects that are not actually present, or rather, are not being accessed by perceptive acts, but by acts of imagination or memory?”
	Verbal thinking	…more or less specific and ordered words and phrases, being experienced privately in the form of one or more voices, recognizable or not, that narrate thoughts, read texts, describe experiences and things, articulate reasoning, enunciate findings and interjections, and/or rehearse conversations that either already took place, are actually happening or could possibly happen?”
	Non-symbolic thought	…thought without clear form, to which pre-reflective, vague and abstract impressions the subject has only intuitive access, as when absorbed/concentrated on a task or event, developing a tacit understanding about something, or sustaining or precipitating some bodily movement, physiological reflex or very fleeting subjective experience?”
	Mind-wandering	…situations in which the subject understands that he/she was not having any conscious experience or that it was devoid of form and sense, and may even feel lost or out of time, as when he/she says that ‘I was wandering’, ‘I don’t know where I was’, ‘I don’t know where I went with my mind’, ‘my mind was a blank/black/blue canvas’, ‘wasn’t thinking/feeling anything’, ‘can’t remember’, ‘wasn’t here’, etc.?”
Sense	Pragmatic	...an experience that is actively sustained and directed towards understanding or eliciting aspects of the external world, such as problem and task solving, building logical reasoning, decision making, and/or planning and time management?”
	Self-regulation	...an experience that is actively sustained and directed towards controlling or eliciting aspects of the self, such as one’s attention, motivation, sensations, affects and affective responses, or the execution and adjustments of reflexes, behavior, and physical movements?
	Self-reflection	...an experience actively sustained and directed towards an epistemic and reflective interest in aspects of the self, whether psychological, spiritual, social, moral, or physical?”
	Observation	...a more or less active and sustained experience, directed towards observation, verification, apprehension, contemplation or appreciation of external aspects or self-aspects?”
Valence	Positive	…an experience occurring in the overall quality of a positive moment, or to affective states and/or sensations communicated exclusively by terms that are easily recognized in common sense as positive?”
	Negative	…an experience occurring in the global quality of a negative moment, or to affective states and/or sensations communicated exclusively by terms that are easily recognized in common sense as negative?”
	Mixed	…an experience occurring in the global quality of an ambivalent moment, or to some affective states and/or sensations communicated by terms that are easily recognized in common sense as positive, and others by terms recognized as negative, simultaneously?”
	Neutral	…an experience occurring in the overall quality of an affectively unimplicated experience, or to affective states and/or sensations communicated by terms that are not easily recognized in common sense as either positive or negative?”

**Table 2:** Sociodemographic characteristics and mental health information. *n* = 64.

	Characteristics	Frequency	Percentage
Age	Below 26 y.o. 26 y.o. and above	25 39	39.1 60.9
Sex	Male Female	23 41	35.9 64.1
Education status	Incomplete higher education degree Complete higher education degree	21 43	32.8 67.2
Area of training	Human Sciences Natural Sciences	47 17	73.4 26.6
Marital status	Single Married Divorced	51 10 3	79.7 15.6 4.7
Children	Yes No	13 51	20.3 79.7
Religion practice	Yes No	25 39	39.1 60.9
Mental diagnosis status	Yes No	11 53	17.2 82.8
Psychotropic use	Yes No	9 55	14.1 85.9
Psychotherapy status	In psychotherapy Have done Never	29 18 17	45.3 28.1 26.6
Meditation practice	Current practice Have done Never	15 26 23	23.4 40.6 35.9
Diary	Current diary Had a diary Never	13 23 28	20.3 35.9 43.8

**Table 3:** Correlations between DSC and SSA variables.

	1	2	3	4	5	6	7	8	9	10
1. PrSC	1									
2. PuSC	0.45 ***	1								
3. Rum	0.04	0.30 *	1							
4. Awa	0.58 ***	0.36 **	−0.22	1						
5. Represent.	−0.37 **	−0.11	−0.19	−0.39 **	1					
6. Public	0.08	0.28 *	0.19	0.16	0.01	1				
7. SelfRegul.	−0.12	−0.39 **	−0.09	0.01	0.16	0.01	1			
8. Verbal	−0.07	−0.14	−0.33*	0.01	0.32 *	0.37 **	0.14	1		
9. Affect	0.10	0.15	−0.03	0.31 *	−0.11	0.08	0.22 *	−0.24	1	
10. Positive	0.09	0.08	−0.07	0.28 *	−0.18	0.02	0.28 *	−0.10	0.77 ***	1
11. Neutral	−0.04	−0.06	0.05	−0.31 *	0.21	0.01	−0.35 **	0.22	−0.92 ***	−0.82 ***

Note: *n* = 64. Items 1–4 = DSC, Items 5–11 = SSA. * *p* < 0.05, ** *p* < 0.01, *** *p* < 0.001.

**Table 4:** Comparisons between awareness DSC subgroups for SSA indexes.

	Low Awa (*n* = 33)	High Awa (*n* = 31)				
	Mean Rank	Mean Rank	*U*	*z*	*p*	Effect Size (*r*)
Affect	26.83	38.53	324.000	−2.51	0.012	−0.367
Positive	26.76	38.61	321.000	−2.55	0.011	−0.372
Representation	39.03	25.55	726.500	−2.90	0.004	0.420
Neutral	38.45	26.16	708.000	−2.64	0.008	0.384

Note: Mann–Whitney *U* test.

## Data Availability

Data can be accessed at https://osf.io/ehafq/?view_only=e6029e964ace4c4cb5341f26f1e05edb, accessed on 25 January 2023.
